# A concept analysis on the transfer climate in health sciences education

**DOI:** 10.1016/j.heliyon.2023.e14299

**Published:** 2023-03-08

**Authors:** Lizemari Hugo-Van Dyk, Yvonne Botma, Mercy Ndhlovu, Champion N. Nyoni

**Affiliations:** School of Nursing, Faculty of Health Sciences, University of the Free State, Bloemfontein, South Africa

**Keywords:** Transfer climate, Rodgers' evolutionary concept analysis, Health sciences education

## Abstract

The transfer of learning is complex, with factors such as transfer climate influencing students' transfer of learning. This transfer climate will shape a student's experiences during work-integrated learning and can be modified to enhance the transfer of learning. However, studies on transfer climate are mainly reported from a human resource development context and the outcomes may not be transferable to health sciences education. Furthermore, there is no uniformity in defining and measuring transfer climate. Rodgers' evolutionary concept analysis approach was used to describe the antecedents, attributes, and consequences of a positive transfer climate. An information specialist assisted in developing a Boolean search string and searched 15 databases to identify relevant sources. In total, 156 relevant articles were selected from 1448 sources. Data were charted and thematically analyzed. Antecedents comprise interpersonal relationships and theory–practice correlation. The presence of student support, training programs, student characteristics, clinical facilitator characteristics and a well-resourced clinical environment are the attributes of a positive transfer climate and act as learning transfer mediators. Transfer climate consequently influences student, educational, and organizational performance. A conceptual definition for transfer climate was then proposed. It was subsequently concluded that developing competent healthcare professionals and providing support to students depend on the synergy and good working relationship between health services and educational institutions. The insights into modifiable elements to enhance transfer climate could benefit health sciences educators in reconsidering their clinical training models to ensure sufficient support during students' clinical placements to meet the demands for a better-qualified healthcare workforce.

## 1 Introduction

The transfer of learning is a complicated process. Studies have demonstrated the challenge of transferring knowledge from one situation to another [1]. The transfer of learning has been defined as a productive application of prior learning and experience in new contexts [[2], [3], [4]]. However, new contexts are potentially different from the original situation in which learning had taken place [5], for example, when health sciences students apply their classroom learning in clinical environments such as a hospital ward. Successful transfer requires recognition of the structure of the abstract concept that lies below the surface details of the problem [6,7]. A significant hindrance for novice learners to transfer their learning is the localization of surface features of specific cases and being oblivious to underlying structural similarities between cases [7]. Kulasegaram et al. [6] showed that students only succeed in recognizing applicability 10–30% of the time when facing new problem scenarios in which previously learned concepts yielded a solution.

The curriculum, the subject matter, the teaching and learning methods, the teaching approach, the student's readiness and willingness to learn, and the environment where the transfer is expected are some of the factors reported to influence students to transfer their learning [8]. The literature describes various hindrances for transfer of learning including cognitive, affective and social barriers within the clinical environment [9]; the pressure to perform which is linked to poor learning habits and a disconnect between what is known and the actual doing in practice [10]. The moment-by-moment learning in a clinical learning environment result from the interaction between individuals, social factors, and ancillary artifacts imbued within the setting, including other students, resources, patient types, clinicians, and educators. This type of learning may be understood as situated learning [11] and is characterized by the critical role of the social setting in which students' acculturation into a new knowledge community enables them to transfer learning. According to the situated learning theory, learning and transfer occur when learners can observe and practice in situ or within authentic learning environments [4].

Health sciences educators understand the crucial role of the learning environment and learning climate in influencing the transfer of learning [12]. As described by Gruppen et al. [4], the learning environment or transfer climate encapsulates social interaction, organizational culture and structures, and physical and virtual spaces that surround and shape students' experiences, perceptions, and transfer of learning. In their explanation of situated learning, Cleland and Durning [13] posit that transfer climate can enhance or be modified to enhance the positive transfer of learning. It is, therefore, vital that health sciences educators modify transfer climate elements to enable students’ positive transfer of learning.

Several studies describe transfer climate elements [4,12,14,15]. However, such studies are predominantly reported from human resource development contexts, and their outcomes may not easily be transferrable to health sciences education. According to Gruppen et al. [4], transfer climate in health sciences education has been subjected to several attempts of description and improvement, with a lack of uniformity in definitions and measuring instruments. The inconsistent and limited literature on the subject creates a challenge for health sciences educators in identifying modifiable variables linked to a supportive transfer climate that enhances the transfer of learning. Therefore, there is a need to define the concept of transfer climate.

Based on the Rodgers’ evolutionary concept analysis approach [16], this article reports on a process of concept analysis to illuminate the antecedents, attributes, and consequences of the concept of transfer of learning in health sciences education. The formulation of a concrete and rigorous definition and enhancement of understanding of transfer climate in health sciences education may foster more relevant, high-quality studies and facilitate educators to enhance the transfer of learning through identifying modifiable elements of transfer climate in health sciences education.

## 2 Method

**Fig. 1:**
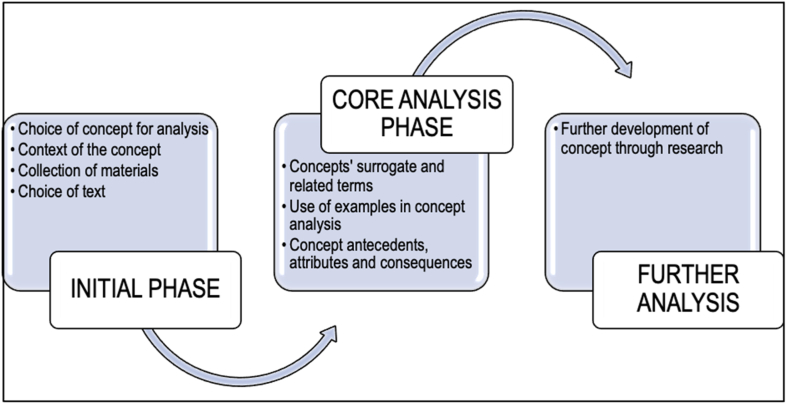
Conceptual framework: Rodgers' evolutionary concept analysis approach (2000).

Concept analysis is a systematic method that allows researchers to examine the characteristics of a concept, consequently providing clarity on a concept that is usually not well defined or one that contributes to confusion [16,17]. Rodgers' evolutionary concept analysis approach was applied, as it acknowledges that concepts develop over time and are influenced by the contexts in which they are used [16]. The concept of transfer climate has been reported predominantly within the human resource development context [2,18]. At the time of the study, no literature could be found in the English language detailing concept analysis of transfer climate in the health sciences education context. In addition, applying Rodgers' evolutionary concept analysis approach provides directions for further research within the context. Methods are presented based on sources of data and the data analysis process. Fig. 1 displays a conceptual framework of Rodgers’ evolutionary concept analysis approach followed in this study.

As described by Tofthagen and colleagues [16], the initial phase of Rogers' evolutionary concept analysis framework is discussed under sources of data. The core analysis phase involves the data extraction and analysis of the attributes, antecedents, and consequences of the concept and the concept's surrogated terms. Further analysis may involve validation of the concept.

### 2.1 Sources of data

An information specialist applied the concept “transfer climate in health sciences education”, integrating Boolean operators and modifiers and appropriate synonyms in searching for relevant articles from 15 databases (see Table 1). The data were searched from CINAHL with full text, MEDLINE with full text, ERIC, Africa-Wide Information, Academic Search Ultimate, SPORTDiscus with full text, PsycINFO, Health Source: Nursing/Academic Edition, Business Source Ultimate, CAB Abstracts, SocINDEX with full text, Communication & Mass Media Complete, Humanities Source, MasterFILE Premier, and Open Dissertations. The authors conducted an ancestry search by examining the references of included articles and expanding the number of articles included in this study. The inclusion criteria were specific to peer-reviewed published articles reflecting on transfer climate and its synonyms for undergraduate students in health sciences that were published in English between 2008 and 2021. The seminal work of Donovan and Darcy [3] on the transfer of learning in human resource development influenced the choice of timelines of this study.

**Table 1:** The search string.

Phenomenon and surrogate terms	“transfer climate” or “clinical learning environment*” or “learning climate*” or “learning culture*” or “organizational climate” or “organizational work climate” or “placement learning” or “psychological climate*” or “practice placement*” or “training climate*” or “transfer environment*” or “work climate*” and “work-integrated learning” AND
Context	“health science*” or “health profession*” or biokinetic* or dentist* or dietetic* or nutrition* or medicine or nursing or “occupational therap*” or optometr* or paramedic* or physiotherapy* or pharmac* or radiograph* or “speech therap*” AND
Population	undergraduate* or student* or resident* or intern or interns or baccalaureate or “higher education” NOT patient* or “school child*” or teacher*

**Fig. 2:**
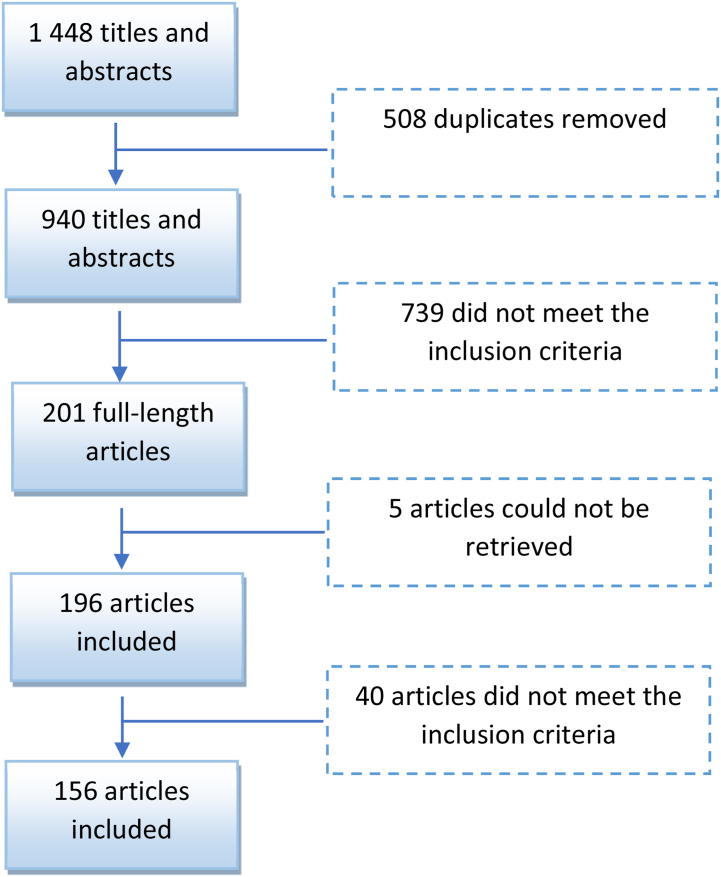
Flow process for inclusion of articles.

The first three authors reviewed the initial 1448 titles and abstracts and exclude 508 titles and abstracts as they were duplicates. The authors further eliminated 739 titles and abstracts that did not meet the inclusion criteria. The full texts were accessed through the information specialist and subjected to the same inclusion criteria. Five articles could not be retrieved and a further 40 full-text articles were eliminated by consensus of the first three authors, resulting in 156 full-text articles included in this study. An ancestry search did not yield any additional articles (see Fig. 2 for the flow process).

### 2.2 Data extraction and analysis process

The author (MN) extracted the study characteristics, including the authors’ names, year of publication, design, and country where the studies were conducted. In addition, the identified antecedents, attributes, and consequences were extracted simultaneously into a Microsoft Excel spreadsheet. The other three authors validated the accuracy of the extracted data by reviewing all the full-text articles and discrepancies were highlighted and resolved through discussion.

An inductive approach through thematic analysis was applied to analyze the extracted codes under the headings of antecedents, attributes, and consequences. The categories and themes in each heading were presented in tables.

## 3 Results

The majority of the articles included in this study were published in 2014, 2015 and 2016, with 21, 18 and 19 articles published, respectively. Study designs used included mainly mixed methods, quantitative studies, and qualitative studies, with the latter being the most used. Most studies were reported from high-income countries.

The outcome of concept analysis should identify a common understanding of the concept of focus within the prescribed context [16]. In this case, the foci of the outcome of this study were elements of the concept, a concretized definition of ‘transfer climate in health sciences education’, and some directions for future research in the field. The surrogate terms of this study are found in Table 1.

### 3.1 Elements of the concept

The elements of the concepts are presented related to the antecedents, attributes, and consequences. In each case, a table with the main themes is set out, with an indication of the number of sources per code. The number next to the category and themes reflects the total sources in the respective category or themes. A discussion of each element follows.

#### 3.1.1 Antecedents

Antecedents relate to events or incidences essential for a concept [16]. Table 2 shows two main antecedents of transfer climate: personal and interpersonal relationships and theory–practice correlation.

**Table 2:** Antecedents of transfer climate in health sciences education.

Themes	Category	Codes	Number of sources
Personal and interpersonal relationships (222)	Attitudes (94)	Motivation of students	33
		A welcoming and respectful environment	30
		Positive staff attitudes and accountability by supervisors and staff	12
		Positive attitudes of students and peer mentors	7
		Acceptance and patience	5
		Staff motivation and motivation by organizations	3
		Positivity and competence of all stakeholders	3
		Confidence in teaching	1
	Collaboration (86)	Trust relationships with educators, staff, and peers (including positive, respectful and nurturing relationships)	42
		Stakeholder collaboration	16
		Shared and established values and valuing students	7
		Responsibility of everyone to support and a community of learning and practice	6
		Availability of educators, professional background of supervisors, and multiple supervisors	5
		Shared ownership of responsibilities	4
		Shared leadership and educational leadership	4
		Commitment from both educators and students	2
	Communication (42)	Effective communicator/Communication	41
		Language of supervisors	1
Theory–practice correlation (107)	Learning opportunities (63)	Available learning and practice opportunities	63
	Student preparedness (44)	Simulation	6
		Educators' responsibility to use learning content, learning materials, simulate learning, continuity of learning, and training documents	5
		Preparation of students (includes orientation before clinical placement, pre-visit to placement area, and orientation)	12
		Prior knowledge (including pre-existing skills)	9
		Preparing students/Student preparedness	4
		Infrastructure, equipment, resources and clinical placement ‘fitness’ for students	1
		Standards of procedure	3
		Adequate classroom teaching	2
		Knowledge sharing	1
		Preparation by manager	1

Personal and interpersonal relationships encompass attitudes of people working in the environment, collaborations in the clinical area, and communication, which should take place during students' clinical learning [19,20]. Stakeholders must ensure that students’ learning environment is favorable for them to transfer learning by portraying good attitudes, acceptance, and mutual respect [19,21,22]. The clinical team, which forms part of the stakeholder group, includes educators who should be welcoming [21,22], clinical staff who are motivated to help students [21], and peers who mentor one another [22,23]. All stakeholders should be positive and display confidence when teaching students [24]. Educators should assist in fostering an environment of collaboration and commitment to student learning [25,26] and should ensure that all stakeholders collaborate and assume leadership roles [27,28].

The collaboration category refers to the basis of trust of all stakeholders, including patients [29]. Clinical staff and facilitators should value students [22], engage in clinical facilitation of students as a shared responsibility, and ensure continuity of learning [20,27]. Communication on information about students’ learning to the clinical placements should be a priority of educational institutions [22]. All stakeholders involved in student learning must communicate among themselves and with students [30,31] using appropriate language [32].

The antecedents related to theory–practice correlation should be considered when planning work-integrated learning, because the placement areas must align with the educational and clinical learning outcomes [33]. Educators or clinical facilitators must be present to assist students during clinical activities [20,34] by orienting students to the clinical practice environment and stimulating prior knowledge [22,35], thereby creating a supportive learning atmosphere [28]. Clinical facilitators must know the students' learning content, be knowledgeable about what they teach, and be available during practice [22,27] to allocate clinical tasks according to each individual's knowledge and skills level [36] for learning transfer during active engagement with patients [37,38].

#### 3.1.2 Attributes

Attributes are constituent elements of a concept that allow for differentiation between concepts to enhance deeper understanding [39]. The attributes of transfer climate are student support, clinical facilitator characteristics, student characteristics, training programs, and environmental resources and characteristics, as set out in Table 3.

**Table 3:** Attributes of transfer climate in health sciences education.

Theme	Categories	Codes	Number of sources
Student support (228)	Supporters (211)	Clinical facilitators (including preceptors, clinical instructors, mentors, clinical teachers, clinical educators, teaching staff, nurse teachers and supervisors)	70
		Clinical staff (including clinicians, clinical practitioners, professional nurses, clinical nurses, staff nurses, registered nurses and other experts in the field)	38
		Peers (including co-workers)	32
		Health system (organizational)	27
		Stakeholders (including interprofessional support and managers, line managers, and clinical team)	22
		Educators (including lecturers, teaching staff and academic staff)	20
		Other people	2
	Types (17)	Social	4
		Learning, teaching	4
		Clinical and skills acquisition	3
		System	3
		Emotional and moral	2
		Cognitive	1
Clinical facilitator characteristics (104)	Professional (62)	Role model	26
		Competent, expert, experienced and knowledgeable	16
		Trained (including trained on teaching techniques to facilitate thinking)	11
		Preparedness and effectiveness	3
		Fairness in grading	3
		Responsibility of student learning	3
	Personal (42)	Availability	8
		Building positive relationships, preceptor–student relationship, understanding students, and supportive partnership	13
		Communication feedback and trust	13
		Social inclusion and socialization tactics	3
		Caring behavior	3
		Personality and character	2
Student characteristics (97)	Self-directedness (47)	Students' effort (including initiative, ownership, responsibility, motivation and contribution) in their learning	14
		Self-evaluation (reflection, self-perception)	10
		Active attendance and participation	8
		Open-mindedness (including openness to learning)	7
		Setting goals (including personal goals and personal outcomes)	4
		Students' accountability as adult learners and making use of clinical practice	3
		Drive	1
	Kinship (41)	Belongingness (including sense of belonging and feeling part of team)	31
		Student–student relationships and learner friendliness	4
		Personal attention (one-on-one)	6
	Self-efficacy (9)	Belief in self (including self-efficacy and own ability)	7
		Confidence	1
		Valuing self as learner	1
Training programs (69)	Curriculum design (48)	Relevance (including curriculum, study material, themes and content, placement duration, adequate time in clinical facilities, clinical exposure, and competence of students according to year of study)	28
		Outcomes (including clinical objective and learning goals)	9
		Student-centered	6
		Availability of learning material, course content, curriculum design, and learning approach	5
	Quality assurance (21)	Knowledgeable supervisor, competent clinicians or educators, mentor knowledge, clinical leader, and clinical faculty	10
		Lecturer expertise and competent and knowledgeable educators	4
		Monitoring and evaluation/grading system	4
		Quality of instruction and program (clear)	3
Environmental resources and characteristics (60)	Resources (32)	Stock (including equipment, supplies, physical material, and consumables)	13
		Human	9
		Resources in general	5
		Facilities	3
		Financial	2
	Positive culture (18)	Workplace culture, ward culture, organizational culture, openness to change, positive atmosphere, supportive culture, positive learning culture, humanistic environment, pedagogical atmosphere, safe space, mastery climate, and permissive atmosphere	13
		Leadership style	5
	Governance structure (10)	Time to complete a task and task completion	4
		Documentation clear and done promptly	3
		Good hospital system	2
		Conflict resolution	1

Student support is necessary for the transfer of learning. Supporters such as clinical facilitators, peers, clinical staff, supervisors, stakeholders, and educators are ancillary to transfer climate in health sciences education. The health system must reflect the different types of student support, such as social, learning, clinical, emotional, system, and cognitive support [40,41].

Clinical facilitators are an essential attribute for transfer climate. According to the literature reviewed in this study, the clinical facilitators are expected to be professional, be role models for the students, possess sufficient competence in their subject area, be prepared and effective in facilitating learning, and be formally trained as facilitators [25,42]. In addition, the facilitators should be available, build positive relationships, communicate effectively, and demonstrate caring behaviors [43].

Student characteristics were divided into three categories: self-directedness, kinship, and self-efficacy. Students’ effort in learning and active attendance and participation are fundamental to self-directness [44,45]. Additional student characteristics linked with self-directedness include open-mindedness, setting goals, student accountability, self-evaluation, and drive [44,45]. Kinship embraces concepts such as belongingness, relationships with peers, and personal attention. Interestingly, the included studies also reflected on self-efficacy in the form of a unique belief system, confidence, and self-value as essential aspects of student characteristics attributable to transfer climate in health sciences education [46].

The training program hinges on curriculum design and quality assurance. The curriculum should be relevant to enhance the transfer of learning [20]. The content, study material, themes, placement duration, time in the clinical setting, and expected competence of student year level should be available and aligned with the environment where the transfer of learning is expected. Authors expected the design and delivery of the training program to be student-centered [47]. Monitoring and evaluation should be embedded in the training program as quality assurance strategies that ensure that clinical facilitators are knowledgeable and competent, and have the necessary expertise [42].

As established, transfer climate in health sciences education is predominantly situated within the clinical environment. The literature reports three aspects of the clinical setting that are essential attributes for transfer climate, namely resources, positive culture, and governance structure. Specific to resources, supplies, including human and financial resources, must be present to support the learning environment [35,48,49]. The workplace work culture and leadership style are expected to be a positive, permanent feature of transfer climate [28,50]. The governance structure and leadership styles of managers that are focused on excellent healthcare systems and conflict resolution are additional attributes of the concept of transfer climate in health sciences education [28,51].

#### 3.1.3 Consequences

Consequences are the results and outcomes of the concept [16]. The consequences of transfer climate in health sciences education are student performance, organizational performance, and educational performance, as displayed in Table 4.

**Table 4:** Consequences of transfer climate in health sciences education.

themes	Category	Codes	Number of sources
Student performance (453)	Competence (142)	Increased competence/performance	62
		Decreased anxiety	1
		Increased satisfaction and achievement	25
		Autonomy and independence	14
		Expertise/Proficiency/Experience	29
		Empowerment	7
		Fit for practice	1
		Decreased time on tasks	3
	Skill (82)	Skill increase (including core, clinical, practice, nursing and learning)	72
		Psychomotor	5
		Interpersonal (including teamwork)	5
	Professionalism (75)	Increased professionalism (including professional identity)	50
		Socialization	17
		Self-directed learning	5
		Moral competence	2
		Lifelong learning	1
	Knowledge (68)	Develop knowledge	68
	Attitude (49)	Increased responsibility	1
		Change in behavior	3
		Commitment	1
		Excitement/Enthusiasm	2
		Empathy	1
		Confidence	41
	Thinking operations (37)	Thinking (including critical thinking, clinical reasoning, clinical judgement and reflective thinking)	27
		Problem solving and decision-making	10
Organizational performance (31)	Better patient care and outcomes (22)	Quality patient care (including patient safety)	22
	Staff (7)	Psychologically safe environment	4
		Staff retainment	2
		Value-sensitive	1
	Financial outcomes (2)	Return on investment	2
Educational performance (27)	High-quality performance (27)	Programme outcomes (including learning outcomes)	14
		Variety of opportunities	13

The consequences of a conducive transfer climate impact student performance [52]. Students gain experience in relating previous tasks to the new practice [34,38], improving their clinical skills, and spending less time per task [42,53]. Furthermore, by becoming critical [29,41], students make better decisions [51] and become more independent [35] while contributing to care [35,54]. Clinical reasoning skills are applied [54], which helps them to become reflective practitioners [55] with appropriate clinical judgment [34].

Transfer climate should result in competent and proficient students who perform expected tasks [35,54]. As students become more competent, supervisors allow them to practice more independently [43,56], which is empowering and quintessential for students to be fit for practice [56].

Social interaction between students and stakeholders in an enabling transfer climate [29,30] enhances self-directedness [44] and develops a culture of lifelong learning [57]. Professional development results in students having a professional identity, thereby maintaining professionalism [37,29,56].

Students develop positive attitudes during clinical practice, which leads to excitement and enthusiasm when they manage clinical situations [42,58]. Furthermore, students show an increase in their responsibilities as they practice without being supervised [35]. Students should show responsibility in meeting their learning outcomes and practice according to expected standards and accomplish their tasks [27].

A consequence of a positive transfer climate is the improvement of organizational performance. Organizations have a better return on investment as students continue to work in the same organization even after graduation [59]. A competitive edge is created when the organization retains highly competent staff and maintains high care standards. Staff experiences a psychologically safe work environment [58] with higher retention ratios. Furthermore, a positive transfer climate improves patient care and health outcomes [60].

An educational consequence of transfer climate in health sciences education is high-quality education. The aim of health sciences programs is to develop a competent health workforce that positively influences health outcomes [61]. Transfer climate in health sciences education must support high-quality educational programs by providing a variety of opportunities for students to meet the program outcomes.

### 3.2 Definition of transfer climate in health sciences education

The authors examined definitions of transfer climate from some of the included articles (see Table 5).

**Table 5:** Extracted definitions of ‘transfer climate’.

SOURCE	DEFINITION
Botma & MacKenzie, 2016:105	Transfer climate is described as “a mediating variable in the relationship between the organizational context and an individual's job attitude and work environment”.
Botma & MacKenzie, 2016:107	Transfer climate involves the connection of an individual's attitude towards the environment in which they are working, which will facilitate or inhibit how one transfers learning.
Baldwin & Ford (cited in Abed, Mansur & Saleh, 2015:466)	Transfer climate is described as “a general construct that has been used to describe those features of the work environment that directly influence the generalisation and maintenance of knowledge and skills learned during training”.
Peters et al., 2014:157–158	Transfer climate refers to “aspects of the work environment that can affect how one transfers learning such as peer support, supervisor support and reinforcement, and personal outcomes which can be both negative and positive”.
Machin & Forgaty (cited in Peters et al., 2014:157–158)	Transfer climate refers to “aspects of the work environment as an opportunity to practice what has been learnt, reinforcement for applying what has been learnt during training courses, and a range of subtle cues in the work environment that enhance or inhibit transfer”.
Sookhai & Budworth, 2010:261	Transfer climate includes supervisors' support to individuals to apply new knowledge to the workplace and to use positive reinforcement for applying learned skills to encourage the transfer of learning.
James, 2010:134	Transfer climate refers to “the supervisor's support in which trainees are expected to apply skills/knowledge learnt in training back in the workplace”.

After integrating the definitions of transfer climate aligned with the described antecedents, attributes, and consequences, the authors coined the following conceptual definition for transfer climate in health sciences education: *Transfer climate in health sciences education is mediated by student* support*, a well-equipped environment, expert clinical facilitators, a well-designed curriculum and student characteristics, which subsequently influence students’ motivation to transfer their learning.* An example of the concepts is provided as a supplementary file to exemplify essential features of the concept in context.

### 3.3 Direction for future research

Empirical studies that focus on manipulating each of the various attributes of the transfer of learning in health sciences with direct measurement of the transfer of learning outcome should be considered as the next step of research for health sciences educators.

## 4 Discussion

This article provides an in-depth description of the concept of transfer climate by exploring specific elements that distinguish this concept in health sciences education. The authors acknowledge that this concept is sensitive to time, context, and conditions and appreciate the value of Rodgers' evolutionary concept analysis approach. This approach supported the integration of a broader systematic search of the literature and the relevant identification of antecedents, attributes, and consequences. The result of this analysis reveals that transfer climate in health sciences education is a complex, multifaceted construct that enhances the transfer of learning with positive outcomes for student performance and the attainment of learning outcomes and broader organizational goals. When compared to the elements in the learning environment as described through the DREEM questionnaire, all elements of the learning environment are included in the transfer climate except for the ‘training programmes’ which is unique to the transfer climate. The ultimate outcome of learning within health sciences must be the transfer of learning in the learning environment. The elements of transfer climate align with salient aspects of learning outcomes in health professions education and extend beyond the learning environment where the transfer is expected to occur.

Personal and professional relationships and theory–practice correlation were the antecedents to transfer climate. These findings chime with the scoping review of clinical placement models by Nyoni et al. [33] They recommend that education models prioritize the establishment, development, and nurturing of positive relationships among various stakeholders, including students, clinical facilitators, and educational institutions. Positive personal and professional relationships serve as fertile grounds for cultivating a transfer climate that supports the transfer of learning. Such relationships must be fostered among health sciences educational institutions and the environments where the transfer of learning is expected.

Theory–practice correlation is an essential antecedent to transfer climate. Social cognitivists acknowledge that students present with prior knowledge built on experience, social interaction, and outside media influences [62]. Building on previous knowledge requires educators to align the transfer environment with the educational outcomes and the class learning activities. Biggs and Tang [63], who describe the concept of constructive alignment, explain that teaching and learning activities and environments should be aligned with learning outcomes. Placing students in a learning environment that is not aligned with their outcomes or what they have learned in the classroom contributes to a cognitive load that hampers any learning transfer [64].

Student support, clinical facilitator characteristics, student characteristics, training programs, and environmental characteristics were identified as the five attributes of transfer climate in health sciences education. These reported attributes are similar to the characteristics of the transfer of learning as described in other fields [3,15]. These attributes are examples of elements of transfer climate that may be modified by health sciences educators and their various stakeholders in contributing toward a conducive transfer climate.

Several studies already point to the unique stressors engendered by the clinical learning environment among health sciences students [65]. Stress during clinical placements contributes to cognitive load, which further impedes any form of learning or transfer thereof [64]. Therefore, health sciences educators must identify their students' need for support. Being sensitive to students' support needs and deliberately engaging best practice inventories, such as the Dundee Ready Education Environment Measure questionnaire, may assist health sciences educators in modifying transfer climate in health sciences education for students [66]. Tailored support strategies that integrate professional and non-professional support may enhance students’ ability to assimilate the affordances of the clinical learning environment.

Faculty development literature exposes health sciences educators as untrained and often unprepared for academic positions [67]. These educators are employed based on their clinical expertise, with minimal focus on their educational qualities, although there are discrepancies in various contexts. The misconstrued educational theories and principles followed by untrained educators are barriers to the effective transfer of learning. Such limitations may be observed in missed opportunities for student learning, poor facilitation approaches, ineffective feedback practices, flawed assessment approaches, and the general pervasive hostile learning environment reported in some settings [68]. According to O'Brien and Battista [11], students immerse themselves in a community of practice and develop through observation and learning within the authentic environments. The leaders of such communities of practice, such as educators and clinical facilitators, should be exemplary role models who are competent in their approach and have sound educational knowledge [25,42]. Investments in teaching health sciences educators and the clinical facilitators on educational philosophy and principles would positively influence transfer climate. Professional education through formal education programs should be mandated for all those who teach, with additional continuing professional development programs.

Students and the environment from which they are learning exist in a synergetic relationship. On the one hand, demotivated students who are not self-directed and who are not engaged within the social structures of learning may not benefit from the learning environment, while on the other hand, the learning environment may demotivate students and toward their efforts of self-direction [4,44]. Student characteristics may be modifiable by initially focusing on selecting individuals with an appropriate aptitude for health sciences. The massification of higher education has contributed to students who may not be mentally and emotionally ready for challenging training programs such as health sciences [69]. Investigating non-cognitive attributes in students, such as resilience, grit, and mindset, could be a worthwhile investment toward appropriating the relevant students for health sciences. Within the program, continuous efforts of molding and cultivating educationally aligned student characteristics through student-centered activities, allowing students to have a voice in their education, fostering group cohesion, and self-reflective practices, may directly influence transfer climate [42,55].

The training program is another modifiable attribute of transfer climate. Advances in the understanding of the science of learning call for curriculum change in health sciences education [70,71] and the subsequent impact of the Covid-19 pandemic on training programs contributes to health educationalists’ implicit and explicit modification of their training programs. Proponents of the contextual curriculum model underpin their argument with the notion that health sciences programs should be based on contextual realities, the local disease burden, available resources, and evidence-based practice [72,73]. A contextual curriculum allows students to learn in environments where the patient profile in practice matches the clinical scenarios described in classroom exercises. This similarity in the cases catalyzes the transfer of learning [6,7]. Therefore, the design of the training program should be underpinned by contextual realities.

Arguably, resources are crucial to transfer climate. The absence or non-alignment of clinical resources with learning outcomes influences the value of transfer climate in the transfer of learning. The learning environment must have appropriate stock and relevant patient mixes staffed by appropriate human resources. However, health sector financing is often reported as suboptimal in most countries, impacting the available resources for patient care and student learning [74]. Ideally, a conducive transfer climate must be well resourced to enable students to transfer learning. This ideal may be achieved through innovative collaborations among national structures responsible for higher education and health services in developing and adopting relevant healthcare financing models that foster optimal financing, accountable utilization, and quality patient care.

An enabling transfer climate has positive consequences for students, educational programs, and the organizations where students transfer their learning. The studies included in this review describe a complex interaction and combination of attributes that contribute to an enabling transfer climate that results in the transfer of learning. A flexible and malleable transfer climate is crucial in grounding students’ acculturation to the communities of practice developed within the clinical learning environment. Such communities may support students, contributing to educational programs and positive organizational outcomes.

A definition of transfer climate in health sciences is proposed, considering the state of the concept within the health sciences context. The authors acknowledge the complexity of this construct and support the argument that transfer climate involves other aspects beyond the environment where the transfer of learning occurs. This work contributes to the literature on the transfer of learning in identifying antecedents, attributes, and consequences of transfer climate in health sciences.

## 5 Conclusion

The transfer of learning is an expected learning outcome for any training or educational program.^5.^ Health sciences students are placed in a clinical learning environment such as teaching hospitals to engage with the community of practice and to learn and transfer their learning. The concept of transfer climate in health sciences was clarified through a concept analysis underpinned by Rodgers' evolutionary concept analysis model, acknowledging that concepts evolve and are context-sensitive [14]. The article presented the antecedents, attributes, and consequences of transfer climate in health sciences and a concise definition of the concept. Insights into modifiable elements to enhance transfer climate could benefit health sciences educators in reconsidering their clinical training models to ensure sufficient support during students’ clinical placements to meet the demands for a better-qualified healthcare workforce. The development of a competent healthcare workforce results from the synergistic interaction between educational institutions and the organizations where students are placed, who collaboratively manipulate elements of transfer climate to enhance learning. Students who rotate through a placement with a positive transfer climate will be motivated to transfer their learning.

The authors acknowledge the following limitations that include the search string, the consulted databases, and the inclusion criteria may have contributed to several limitations within this analysis. The bias towards English language publications may have skewed the outcomes of this study. Exploring this concept in non-English and non-traditional health sciences contexts such as allopathic medicine is a possible recommendation from this study.

## Declarations

### Author contribution statement

Lizemari Hugo-van Dyk; Yvonne Botma: Conceived and designed the experiments; Performed the experiments; Analyzed and interpreted the data; Contributed reagents, materials, analysis tools or data; Wrote the paper.Mercy Ndhlovu: Performed the experiments; Analyzed and interpreted the data; Contributed reagents, materials, analysis tools or data; Wrote the paper.Champion Nyoni: Analyzed and interpreted the data; Contributed reagents, materials, analysis tools or data; Wrote the paper.

### Funding statement

This research did not receive any specific grant from funding agencies in the public, commercial, or not-for-profit sectors.

### Data availability statement

Data will be made available on request.

### Declaration of interest's statement

The authors declare no conflict of interest.

## References

[1] Laksov K.B., Lonka K., Josephson A. (2008). How do medical teachers address the problem of transfer?. Adv. Health Sci. Educ..

[2] Holton E.F., Bates R.A., Ruona W.E.A. (2000). Development of a generalized learning transfer system inventory. Hum. Resour. Dev. Q..

[3] Donovan P., Darcy D.P. (2011). Learning transfer: the views of practitioners in Ireland. Int. J. Train. Dev..

[4] Gruppen L.D., Irby D.M., Durning S.J., Maggio L.A. (2019). Conceptualizing learning environments in the health professions. Acad. Med..

[5] Hajian S. (2019). Transfer of learning and teaching: a review of transfer theories and effective instructional practices. IAFOR J Educ.

[6] Kulasegaram K., Min C., Ames K., Howey E., Neville A., Norman G. (2012). The effect of conceptual and contextual familiarity on transfer performance. Adv. Health Sci. Educ..

[7] Norman G., Dore K., Krebs J., Neville A.J. (2007). The power of the plural: effect of conceptual analogies on successful transfer. Acad. Med..

[8] Augustsson H., Törnquist A., Hasson H. (2013). J. Health Organisat. Manag..

[9] Kahlke R.M., McConnell M.M., Wisener K.M., Eva K.W. (2020 Mar). The disconnect between knowing and doing in health professions education and practice. Adv. Health Sci. Educ..

[10] Melvin L., Rassos J., Panisko D., Driessen E., Kulasegaram K.M., Kuper A. (2019 Feb 1). Overshadowed by assessment: understanding trainee and supervisor perspectives on the oral case presentation in internal medicine workplace-based assessment. Acad. Med..

[11] O'Brien B.C., Battista A. (2020). Situated learning theory in health professions education research: a scoping review. Adv. Health Sci. Educ..

[12] Jackson C.B., Brabson L.A., Quetsch L.B., Herschell A.D. (2019). Training transfer: a systematic review of the impact of inner setting factors. Adv. Health Sci. Educ..

[13] Cleland J., Durning S.J. (2019). Education and service: how theories can help in understanding tensions. Med. Educ..

[14] Hughes A.M., Zajac S., Spencer J.M., Salas E. (2018 Dec 1). A checklist for facilitating training transfer in organizations. Int. J. Train. Dev..

[15] Baldwin T.T., Ford J.K. (1988 Mar). Transfer of training: a review and directions for future research. Person. Psychol..

[16] Tofthagen R., Fagerstrøm L.M. (2010 Dec). Rodgers' evolutionary concept analysis a valid method for developing knowledge in nursing science. Scand. J. Caring Sci..

[17] Brush B.L., Kirk K., Gultekin L., Baiardi J.M. (2011 Jul 1). Overcoming: a concept analysis. Nurs. Forum.

[18] Brion C. (2022 Apr). Culture: the link to learning transfer. Adult Learn..

[19] Williamson G.R., Plowright H., Kane A., Bunce J., Clarke D., Jamison C. (2020 Feb 1). Collaborative learning in practice: a systematic review and narrative synthesis of the research evidence in nurse education. Nurse Educ. Pract..

[20] Muthathi I.S., Thurling C.H., Armstrong S.J. (2017). Through the eyes of the student: best practices in clinical facilitation. Curationis.

[21] Metzger M., Dowling T., Guinn J., Wilson D.T. (2020 Jan 1). Inclusivity in baccalaureate nursing education: a scoping study. J. Prof. Nurs..

[22] Brady M., Price J., Bolland R., Finnerty G. (2019). Needing to belong: first practice placement experiences of children's nursing students. Compr Child Adolesc Nurs.

[23] McPake M. (2021 Feb 1). How do the attitudes of therapeutic radiographers affect students' learning during practice placement?. Radiography.

[24] du Plessis J., Bezuidenhout J. (2019 Nov 12). Areas of good practice and areas for improvement in work-integrated learning for radiography training in South Africa. Afr J Health Prof Educ.

[25] Weber K., Carter B., Jenkins G., Jamieson J. (2019 Sep). A dietetic clinical educator enhances the experience and assessment of clinical placement. Nutr. Diet..

[26] Rajeswaran L. (2017). Clinical experiences of nursing students at a selected institute of health sciences in Botswana. Health Sci. J..

[27] Peters S., Clarebout G., van Nuland M., Aertgeerts B., Roex A. (2018). A qualitative exploration of multiple perspectives on transfer of learning between classroom and clinical workplace. Teach. Learn. Med..

[28] Gurková E., Ziaková K. (2018). Evaluation of the clinical learning experience of nursing students: a cross-sectional descriptive study. Int. J. Nurs. Educ. Scholarsh..

[29] Hugo L. (2018). https://scholar.ufs.ac.za/handle/11660/9879.

[30] Materne M., Henderson A., Eaton E. (2017). Building workplace social capital: a longitudinal study of student nurses' clinical placement experiences. Nurse Educ. Pract..

[31] DiMattio M.J., Hudacek S.S. (2020 Nov 1). Educating generation Z: psychosocial dimensions of the clinical learning environment that predict student satisfaction. Nurse Educ. Pract..

[32] Sookhai F., Budworth M.H. (2010 Sep 1). The trainee in context: examining the relationship between self-efficacy and transfer climate for transfer of training. Hum. Resour. Dev. Q..

[33] Nyoni C.N., Hugo-van Dyk L., Botma Y. (2021 Dec 4). Clinical placement models for undergraduate health professions students: a scoping review. BMC Med. Educ..

[34] Botma Y., MacKenzie M.J. (2016). Perspectives on transfer of learning by nursing students in primary healthcare facilities. J. Nurse Educ. Pract..

[35] Bawadi H.A., Al-Hamdan Z.M., Nabolsi M., Abu-Moghli F., Zumot A., Walsh A. (2019 Jan 1). Jordanian nursing student and instructor perceptions of the clinical learning environment. Int. J. Nurs. Educ. Scholarsh..

[36] Kgafele N.S., Coetzee I., Heyns T. (2015). Clinical accompaniment let the voice of the pre-graduate students count. Afr J Nurs Midwifery.

[37] Galletta M., Portoghese I., Alviles-Gonzales C., Melis P., Marcias G., Campagna M. (2017). Lack of respect, role uncertainty and satisfaction with clinical practice among nursing students: the moderating role of supportive staff. Acta Biomed Atenai Parm.

[38] Al-Osaimi D.N., Fawaz M. (2022 Jul 1). Nursing students' perceptions on motivation strategies to enhance academic achievement through blended learning: a qualitative study. Heliyon.

[39] Shahsavari H., Zarei M., Aliheydari Mamaghani J. (2019 Nov 1). Transitional care: concept analysis using Rodgers' evolutionary approach. Int. J. Nurs. Stud..

[40] Hugo-Van Dyk L., Botma Y., Raubenheimer J.E. (2022 Jan 1). Confirmation of an instrument monitoring quality of nursing student accompaniment. Int J Africa Nurs Sci.

[41] Henderson A., Harrison P., Rowe J., Edwards S., Barnes M., Henderson S. (2018;31(April). Students take the lead for learning in practice: a process for building self-efficacy into undergraduate nursing education. Nurse Educ. Pract..

[42] Baird K., Hastie C.R., Stanton P., Gamble J. (2022 Feb 1). Learning to be a midwife: midwifery students' experiences of an extended placement within a midwifery group practice. Women Birth.

[43] McQueen K.A., Poole K., Raynak A., McQueen A. (2018). Preceptorship in a nurse practitioner program: the student perspective. Nurse Educat..

[44] Wong F.M., Tang A.C., Cheng W.L. (2021 Sep 1). Factors associated with self-directed learning among undergraduate nursing students: a systematic review. Nurse Educ. Today.

[45] Brandt W.C. (2020). http://creativecommons.org/licenses/by/4.0/.

[46] Daly M., Salamonson Y., Glew P.J., Everett B. (2017 Oct 1). Hawks and doves: the influence of nurse assessor stringency and leniency on pass grades in clinical skills assessments. Collegian.

[47] Rathore E., Riaz F., Habib N., Anjum O., Zahra R., Salahuddin M.B. (2022 Oct 4). A comparison between teacher centered and student centered medical education approach: an experimental research. Pakistan J Medical Health Sci.

[48] Motsaanaka M.N., Makhene A., Ally H. (2020). Student nurses' experiences regarding their clinical learning opportunities in a public academic hospital in Gauteng province, South Africa. Health SA Gesondheid.

[49] Rahimi M., Haghani F., Kohan S., Shirani M. (2019 Dec 1). The clinical learning environment of a maternity ward: a qualitative study. Women Birth.

[50] Arundell F., Mannix J., Sheehan A., Peters K. (2018). Workplace culture and the practice experience of midwifery students: a meta-synthesis. J. Nurs. Manag..

[51] Antohe I., Riklikiene O., Tichelaar E., Saarikoski M. (2016 Mar 1). Clinical education and training of student nurses in four moderately new European Union countries: assessment of students' satisfaction with the learning environment. Nurse Educ. Pract..

[52] Barry S., Martin C. (2018). Factors impacting on the success of clinical learning - a student and nurse educator perspective. Aust. Nurs. Midwifery J..

[53] Brandford E., Hasty B., Bruce J.S., Merrell S.B., Shipper E.S., Lin D.T., Lau J.N. (2018 Feb 1). Underlying mechanisms of mistreatment in the surgical learning environment: a thematic analysis of medical student perceptions. Am. J. Surg..

[54] Norouzi N., Imani B. (2021 Apr). Clinical education stressors in operating room students: a qualitative study. Invest. Educ. Enfermería.

[55] Soler O.M., Aguayo-González M., Gutiérrez S.S., Pera M.J., Leyva-Moral J.M. (2021 Mar 1). Nursing students' expectations of their first clinical placement: a qualitative study. Nurse Educ. Today.

[56] Thomson R., Docherty A., Duffy R. (2017 May 11). Nursing students experience of mentorship in their final placement. Br. J. Nurs..

[57] Plack M.M., Goldman E.F., Wesner M., Manikoth N., Haywood Y. (2015). How learning transfers: a study of how graduates of a faculty education fellowship influenced the behaviors and practices of their peers and organizations. Acad. Med..

[58] Davis K., Doole E., Cheek C., Shires L. (2019 Oct). How medical students learn in primary care. Clin. Teach..

[59] Hegenbarth M., Rawe S., Murray L., Arnaert A., Chambers-Evans J. (2015). Establishing and maintaining the clinical learning environment for nursing students: a qualitative study. Nurse Educ. Today.

[60] Jonsén E., Melender H.L., Hilli Y. (2013). Finnish and Swedish nursing students' experiences of their first clinical practice placement - a qualitative study. Nurse Educ. Today.

[61] Lawler M.J., Curry D., Donnenwirth J., Mangrich M.E., Times T.N. (2012). Assessing transfer-of-learning potential with human services professionals. J. Soc. Serv. Res..

[62] Taylor D.C.M., Hamdy H. (2013 Nov 4). Adult learning theories: implications for learning and teaching in medical education: AMEE Guide No. 83. Med. Teach..

[63] Biggs J, Tang C. Teaching for Quality Learning at University. fourth ed. New York: McGraw-Hill.

[64] Ginat D., Shifroni E., Menashe E. (2011 Oct 26). Transfer, cognitive load, and program design difficulties. Lect. Notes Comput. Sci..

[65] Gazzaz Z.J., Baig M., Al Alhendi B.S.M., Al Suliman M.M.O., Al Alhendi A.S., Al-Grad M.S.H. (2018 Feb 23). Perceived stress, reasons for and sources of stress among medical students at rabigh medical college, king abdulaziz university, jeddah, Saudi arabia. BMC Med. Educ..

[66] Kim H., Jeon P., Kim S., Hong J., Kang Y. (2021). Cross-cultural adaptation and validation of the Korean version of the dundee ready education environment measure (DREEM). Evid. base Compl. Alternative Med..

[67] Irby D.M. (1996). Models of faculty development for problem-based learning. Adv. Health Sci. Educ..

[68] Kamphinda S., Chilemba E.B. (2019 Jan 1). Clinical supervision and support: perspectives of undergraduate nursing students on their clinical learning environment in Malawi. Curationis.

[69] de Klerk D., Spark L., Jones A., Maleswena T. (2017). Paving the road to success: reflecting critically on year one of an undergraduate student support programme at a large South African university. J Student Aff Africa.

[70] Rich J.V., Fostaty Young S., Donnelly C., Hall A.K., Dagnone J.D., Weersink K. (2020). Competency‐based education calls for programmatic assessment: but what does this look like in practice?. J. Eval. Clin. Pract..

[71] Irby D.M., Cooke M., Brien B.C.O. (2010). Calls for reform of medical education by the carnegie foundation for the advancement of teaching : 1910 and 2010. Acad. Med..

[72] Bates J., Schrewe B., Ellaway R.H., Teunissen P.W., Watling C. (2019 Jan 153). Embracing standardisation and contextualisation in medical education. Med. Educ..

[73] Schrewe B., Ellaway R.H., Watling C., Bates J. (2018). The contextual curriculum: learning in the matrix, learning from the matrix. Acad. Med..

[74] WHO. Global expenditure on health: public spending on the rise?. https://www.who.int/publications/i/item/9789240041219.

