# Supplementary material for: A concept analysis on the transfer climate in health sciences education
Source: Heliyon. 2023 Mar 8;9(3):e14299. doi: 10.1016/j.heliyon.2023.e14299 (PMC10036515; doi:10.1016/j.heliyon.2023.e14299)
Supplement: Multimedia component 1 [file mmc1.docx]

All fourth-year students from all the health science programs at a local university participate in an interprofessional placement on a rural clinical learning platform. Prior to the clinical placements, facilitators from the various programs learned how to facilitate interprofessional student groups to achieve collaborative learning outcomes. Preparatory learning and teaching activities for students included, among others, to developing shared values – including people-centredness; clarifying professional roles; demonstrating shared leadership; and team communication in simulated situations. During the preparatory phase, the interprofessional small groups built trusting relationships and gained an understanding of the other professionals’ roles and scope of practice.

The clinical placements comprised interprofessional student small groups with a clinical facilitator per group to support the students’ learning throughout the placement. On arrival at the clinical learning platform, the site coordinator orientated everyone to the physical layout of the facility and the specific learning outcomes for the placement. The arrangements made with the stakeholders, for example, the principal at the local high school, the manger at the primary healthcare clinic, and others were shared and contact details were provided. Based on assessment findings, the student groups had to plan and implement interventions in collaboration with the relevant stakeholders. Collaborative planning assisted the students in being culturally sensitive, using available resources within governance structures.

On completion of their placement, the student’s self-evaluation and reflection on achieving the learning outcomes were portrayed through collaborative digital storytelling. Most digital stories highlighted the new friendships formed, insight into other health professionals’ scope of practice, and the patients’ harsh reality in rural areas. The interprofessional clinical placement strengthened the students’ health services and educational performance in rural areas.
